# Supplementary material for: Comparison Study of Small Extracellular Vesicle Isolation Methods for Profiling Protein Biomarkers in Breast Cancer Liquid Biopsies
Source: Int J Mol Sci. 2023 Oct 23;24(20):15462. doi: 10.3390/ijms242015462 (PMC10607056; doi:10.3390/ijms242015462)
Supplement: Supplementary file 1 [file ijms-24-15462-s001.zip › ijms-2651930-supplementary.pdf]

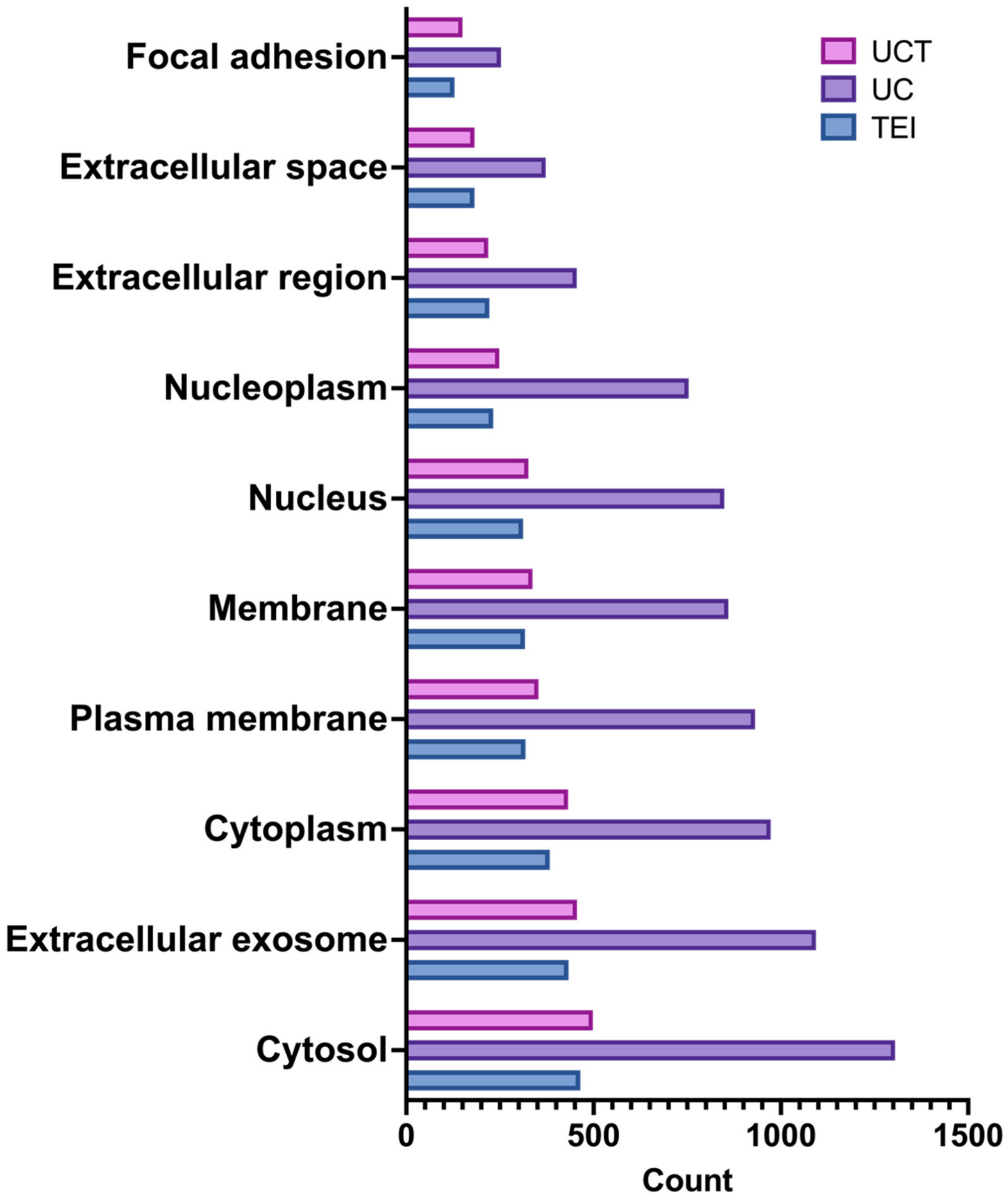

Figure S1. Top 10 most abundant GO analysis term of identified proteins in MDA-MB-231 cell derived sEV isolates from three different isolation methods. Go enrichment analysis of total identified protein isolated by TEI, UC, and UCT, isolation methods were carried out using DAVID. The graph shows 10 most abundant GO cellular component term and its count (P value<0.05, FDR<0.01). **Abbreviations:** TEI: total exosome isolation kit; UC: ultracentrifugation; UCT: ultracentrifugation followed by total exosome isolation kit.

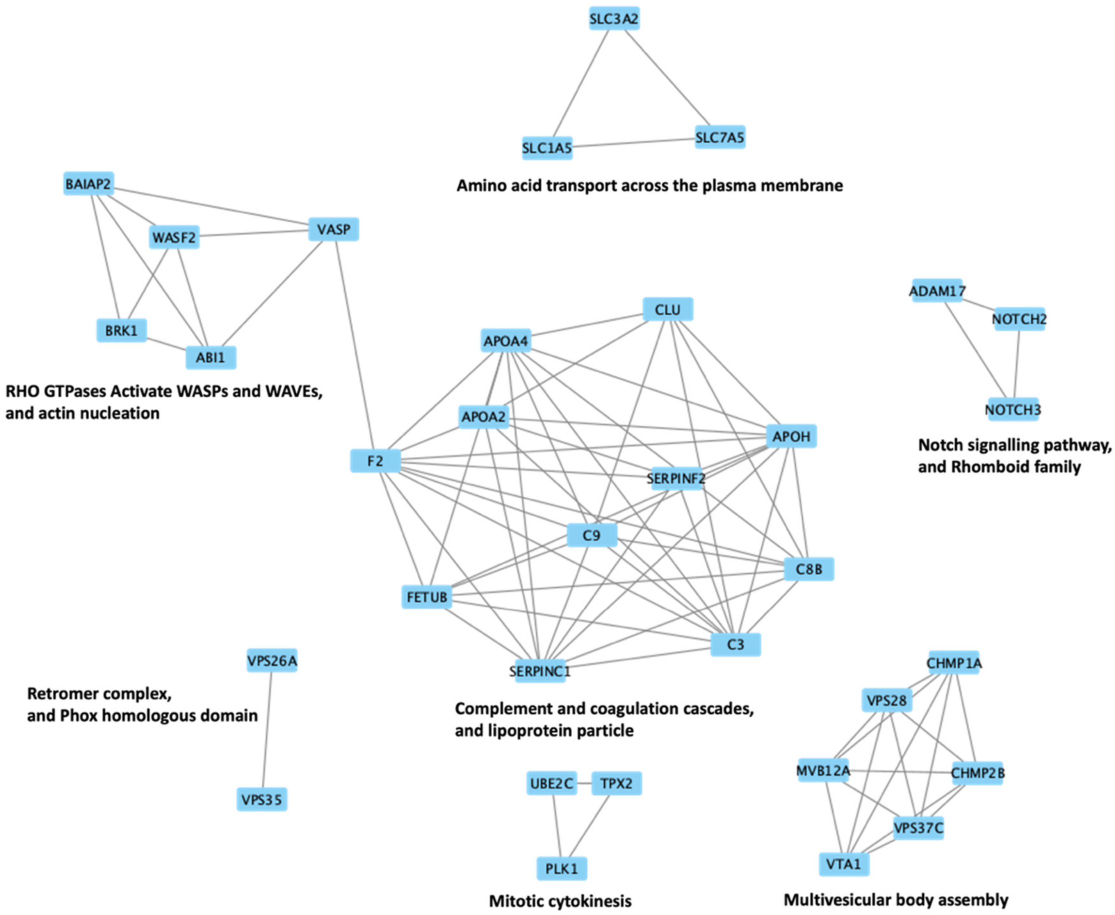

Figure S2. STRING network analysis of upregulated and downregulated sEV proteins in BC cell lines by UC. The figure shows the predicted sEV protein-protein interaction in BC cell lines and each cluster demonstrates its biological function (FDR=0.01).

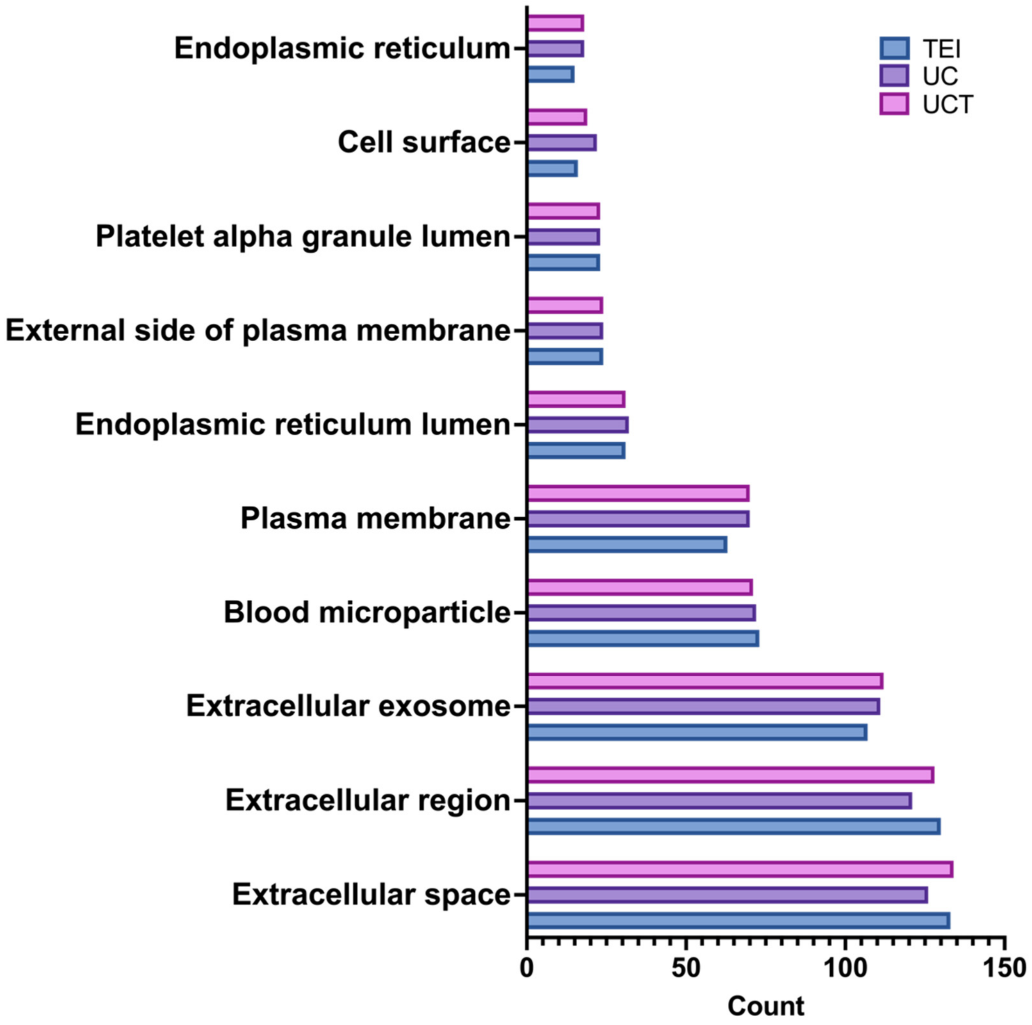

Figure S3. Top 9 most abundant GO analysis term of identified proteins in BC plasma derived sEV isolates from three different isolation methods. Go enrichment analysis of total identified protein isolated by UC, UCT, and TEI isolation methods were carried out using David. The graph shows 9 most abundant GO cellular component term and its count (P <0.05, FDR<0.01). **Abbreviations:** TEI: total exosome isolation kit; UC: ultracentrifugation; UCT: ultracentrifugation followed by total exosome isolation kit.

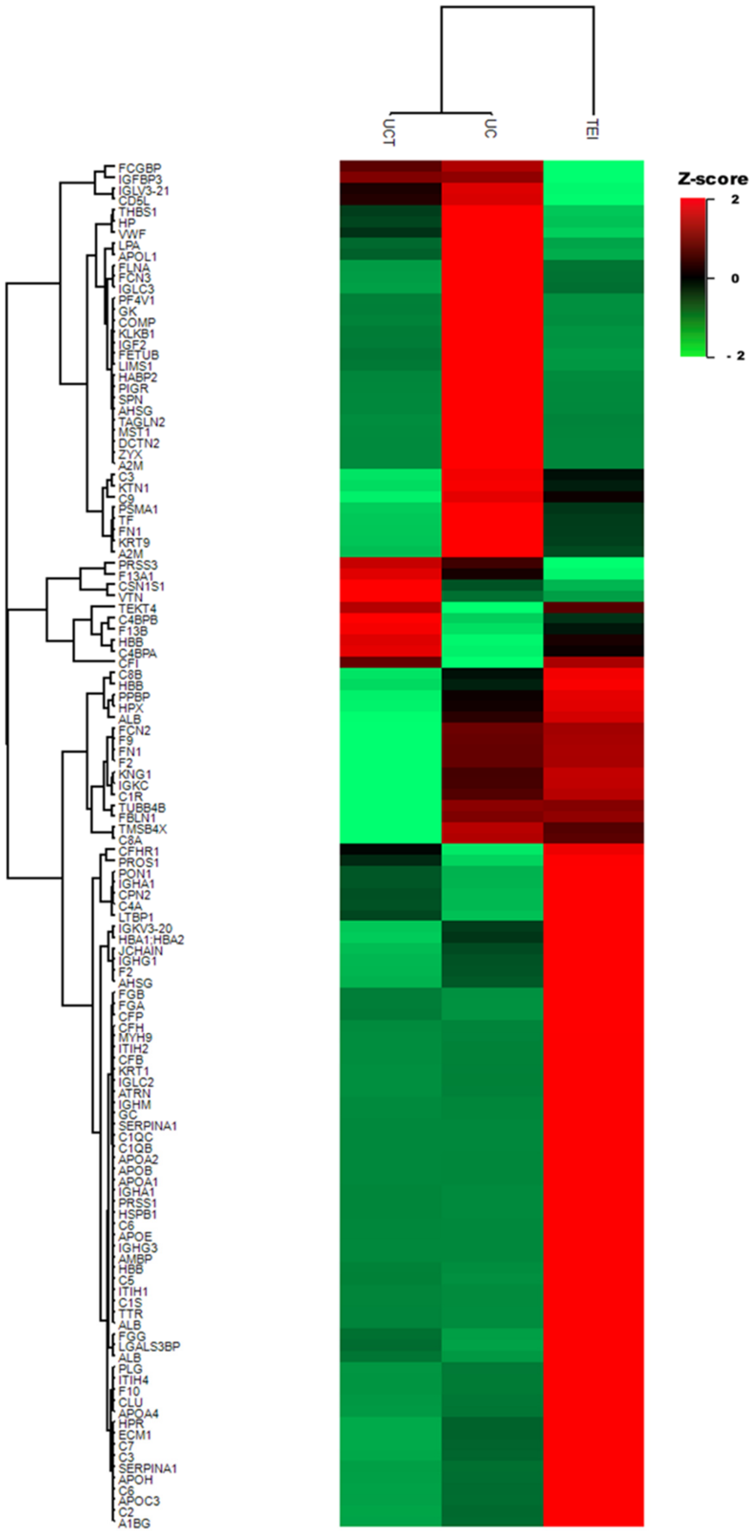

Figure S4. Proteomic profiling of sEV proteins isolated by three different isolation methods in BC plasma. Proteomic profiling of sEV proteins isolated by three different isolation methods in BC plasma.

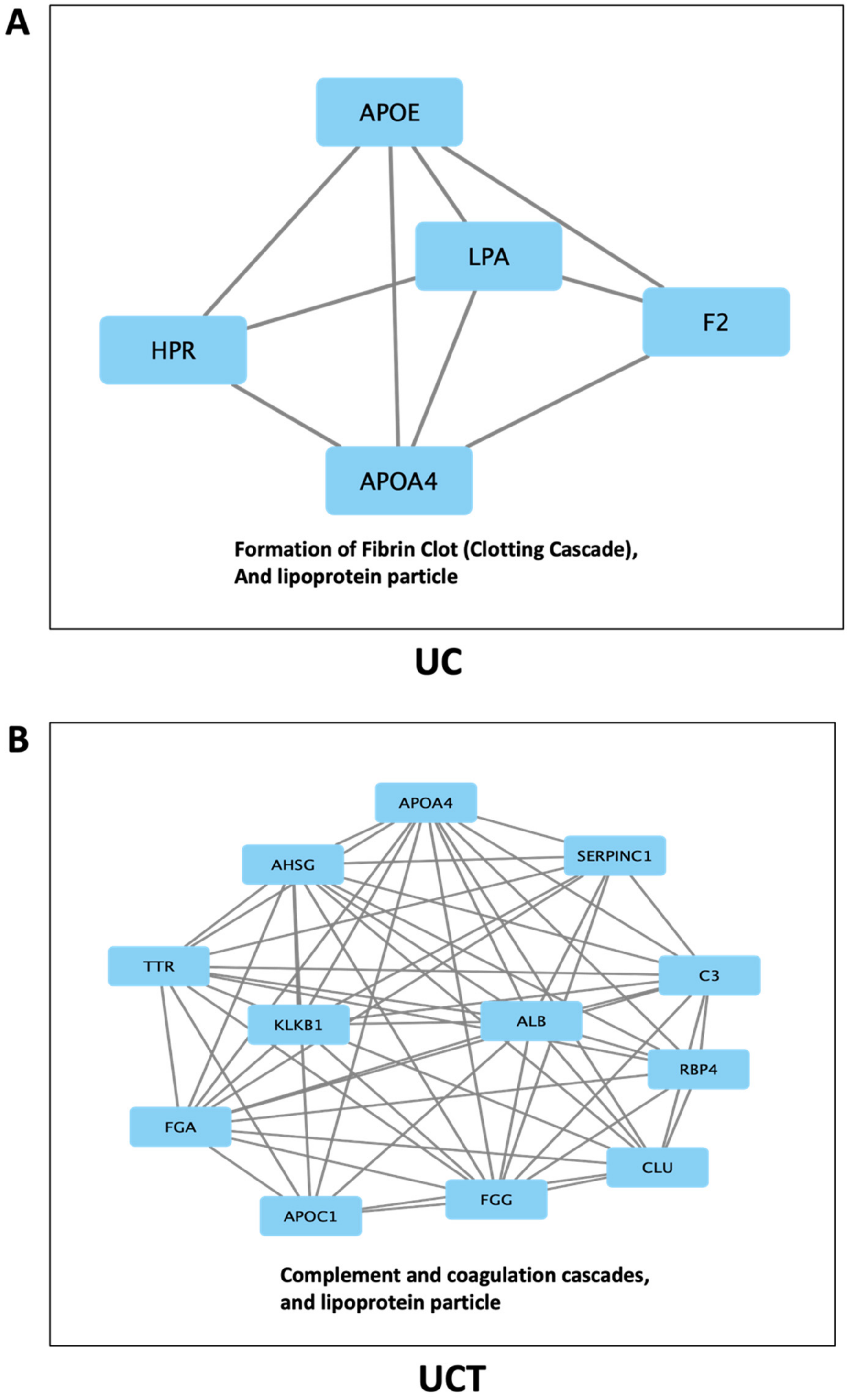

Figure S5. STRING network analysis of upregulated and downregulated sEV proteins in BC plasma isolated by UC and UCT. The figures represent the predicted sEV protein-protein interaction in BC plasma and each cluster demonstrates its biological function (FDR=0.01).

Table S1. Top 10 significant GO terms for cellular component of identified proteins from MDA-MB-231 cell line

**TEI**				
**Cellular component**	**Count**	**Percentage of genes (%)**	***P* value**	**FDR**
Cytosol	465	54.4	1.60E-69	3.60E-67
Extracellular exosome	433	50.7	4.60E-200	3.10E-197
Cytoplasm	383	44.8	5.30E-30	4.40E-28
Plasma membrane	318	37.2	2.80E-14	8.80E-13
Membrane	317	37.1	1.10E-41	1.50E-39
Nucleus	312	36.5	5.40E-07	7.00E-06
Nucleoplasm	232	27.2	3.70E-09	7.50E-08
Extracellular region	222	26	4.40E-41	4.90E-39
Extracellular space	182	21.3	7.00E-27	5.20E-25
Focal adhesion	129	15.1	2.50E-75	8.20E-73
**UC**				
**Cellular component**	**Count**	**Percentage of genes (%)**	***P* value**	**FDR**
Cytosol	1305	51.7	9.80E-185	4.70E-182
Cytoplasm	1094	43.3	6.90E-80	1.30E-77
Extracellular exosome	973	38.5	0.00E+00	0.00E+00
Nucleus	931	36.9	1.20E-21	5.00E-20
Plasma membrane	860	34.1	1.40E-24	6.90E-23
Membrane	849	33.6	1.60E-92	3.70E-90
Nucleoplasm	754	29.9	2.30E-45	3.10E-43

Extracellular region	455	18	3.60E-38	3.80E-36
Extracellular space	372	14.7	4.80E-21	1.80E-19
Focal adhesion	253	10	7.10E-122	2.20E-119
**UCT**				
**Cellular component**	**Count**	**Percentage of genes (%)**	***P* value**	**FDR**
Cytosol	498	55.1	2.30E-76	5.30E-74
Extracellular exosome	456	50.5	1.30E-209	9.10E-207
Cytoplasm	432	47.8	1.30E-41	1.50E-39
Membrane	353	39.1	4.20E-52	7.10E-50
Nucleus	337	37.3	2.40E-08	3.80E-07
Plasma membrane	326	36.1	2.20E-12	5.90E-11
nucleoplasm	248	27.5	5.00E-10	8.50E-09
Extracellular region	219	24.3	2.50E-35	2.20E-33
Extracellular space	182	20.2	9.60E-24	5.50E-22
Focal adhesion	150	16.6	5.40E-95	1.90E-92

Table S2. Lipoprotein related cellular component in MDA-MB-231 cell line derived sEV proteins

**Isolation method**	**Cellular component related to Lipoproteins**	**Count**	**Percentage of genes (%)**	***P* value**	**FDR**
TEI	Chylomicron	7	0.8	1.10E-05	1.10E-04
	Very-low-density lipoprotein	7	0.8	1.50E-04	1.30E-03

	Spherical high-density lipoprotein particle	4	0.5	3.30E-03	1.90E-02
	High-density lipoprotein particle	5	0.6	2.30E-02	9.00E-02
UC	Very-low-density lipoprotein	13	0.5	1.00E-06	9.80E-06
	Chylomicron	10	0.4	6.30E-06	5.30E-05
	High-density lipoprotein particle	12	0.5	1.60E-04	1.00E-03
	Intermediate-density lipoprotein particle	5	0.2	9.70E-04	4.90E-03
	Spherical high-density lipoprotein particle	6	0.2	1.10E-03	5.20E-03
	Low-density lipoprotein particle	6	0.2	1.40E-02	5.00E-02
UCT	Chylomicron	7	0.8	1.50E-05	1.40E-04
	Very-low-density lipoprotein particle	7	0.8	2.10E-04	1.60E-03
	High-density lipoprotein particle	6	0.7	5.70E-03	2.90E-02
	Low-density lipoprotein particle	4	0.4	1.70E-02	7.10E-02

Table S3. The most unique expressed sEV proteins in BC cell lines compared to normal breast cell.

		MDA-MB-231/ Normal breast		MCF-7/ Normal breast		SK-BR-3/ Normal breast	
Gene symbol	Expression in BC cell lines	Abundance Ratio (log2)	P value	Abundance Ratio (log2)	P value	Abundance Ratio (log2)	P value
CEBPZ	Up	9.77	<0.000001	9.97	6.03E-08	9.46	<0.000001
CHMP1A	Up	3.49	0.000565	4.49	2.23E-05	2.73	0.001111
CLTA	Up	7	0.000264	6.99	0.00024545	3.7	0.007280
IFITM1	Up	9.97	0.000020	9.97	2.37E-06	9.97	0.000016
SEC13	Up	7.69	0.000006	8.05	4.87E-06	7.4	0.000013
VTA1	Up	7.87	0.000603	7.96	0.00052427	7.89	0.000703
BDH2	Down	-8.11	0.000717	-8.35	0.00029472	-8.28	0.000312
INS	Down	-9.97	<0.000001	-9.97	4.6098E-07	-9.97	<0.000001
LAMA3	Down	-8.04	0.000318	-9.41	0.00012396	-9.39	0.000095

TPX2	Down	-8.03	0.008227	-9.97	0.0004124<br>4	-8.54	0.001329

Table S4. Top 9 significant GO terms for cellular component of identified proteins from BC patients' plasma.

TEI				
Cellular component	Count	Percentage of genes (%)	P value	FDR
Extracellular space	133	81.1	1.20E-105	1.10E-103
Extracellular region	130	79.3	4.90E-96	3.00E-94
Extracellular exosome	107	65.2	2.50E-62	1.20E-60
Blood microparticle	73	44.5	9.40E-118	1.70E-115
Plasma membrane	63	38.4	4.30E-04	2.40E-03
Endoplasmic reticulum lumen	31	18.9	2.90E-24	8.90E-23
External side of plasma membrane	24	14.6	4.80E-12	6.80E-11
Platelet alpha granule lumen	23	14	2.70E-30	1.00E-28
Cell surface	16	9.8	1.70E-04	1.10E-03
Endoplasmic reticulum	15	9.1	7.00E-02	2.10E-01
**UC**				
Cellular component	Count	Percentage of genes (%)	P value	FDR
Extracellular space	126	71.6	4.00E-88	4.60E-86
Extracellular region	121	68.8	6.70E-77	5.10E-75
Extracellular exosome	111	63.1	2.40E-62	1.30E-60
Plasma membrane	72	40.9	1.50E-05	1.10E-04

Blood microparticle	70	39.8	4.30E-108	9.70E-106
Endoplasmic reticulum Lumen	32	18.2	1.60E-24	6.10E-23
External side of plasma Membrane	24	13.6	2.10E-11	5.40E-10
Platelet alpha granule lumen	23	13.1	1.40E-29	6.30E-28
Cell surface	22	12.5	1.20E-07	1.40E-06
Endoplasmic reticulum	18	10.2	1.80E-02	7.40E-02
**UCT**				
Cellular component	Count	Percentage of genes (%)	P value	FDR
Extracellular space	134	75.7	3.40E-99	3.60E-97
Extracellular region	128	72.3	8.70E-86	6.10E-84
Extracellular exosome	112	63.3	3.90E-63	2.10E-61
Plasma membrane	71	40.1	3.70E-05	2.70E-04
Blood microparticle	70	39.5	7.00E-108	1.50E-105
Endoplasmic reticulum lumen	31	17.5	3.00E-23	1.10E-21
External side of plasma membrane	24	13.6	2.40E-11	5.60E-10
Platelet alpha granule lumen	23	13	1.60E-29	6.70E-28
Cell surface	19	10.7	9.40E-06	7.90E-05
Immunoglobulin complex	18	10.2	6.40E-18	1.90E-16

Table S5. Lipoprotein related cellular component of sEV proteins derived from BC patients' plasma.

Isolation method	Cellular component related to Lipoproteins	Count	Percentage of genes (%)	*P* value	FDR
TEI	Very-low-density lipoprotein particle	9	5.5	2.40E-12	4.10E-11
	High-density lipoprotein particle	9	5.5	2.50E-11	3.10E-10
	Spherical high-density lipoprotein particle	7	4.3	6.20E-12	8.20E-11
	Intermediate-density lipoprotein particle	3	1.8	6.10E-04	3.40E-03
	Low-density lipoprotein particle	3	1.8	4.60E-03	2.20E-02
	Discoidal high-density lipoprotein particle	2	1.2	1.60E-02	6.20E-02
	Chylomicron	7	4.3	6.40E-10	7.00E-09
UC	Very-low-density lipoprotein particle	8	4.5	3.00E-10	5.20E-09
	High-density lipoprotein particle	8	4.5	2.20E-09	3.10E-08
	Spherical high-density lipoprotein particle	6	3.4	2.30E-09	3.10E-08
	Intermediate-density lipoprotein particle	3	1.7	7.10E-04	4.10E-03
	Chylomicron	7	4	9.80E-10	1.50E-08

UCT	Very-low-density lipoprotein particle	8	4.5	3.10E-10	5.50E-09
	High-density lipoprotein particle	8	4.5	2.30E-09	2.90E-08
	Spherical high-density lipoprotein particle	6	3.4	2.40E-09	2.90E-08
	Intermediate-density lipoprotein particle	3	1.7	7.10E-04	4.10E-03
	Chylomicron	7	4	1.00E-09	1.40E-08

Table S6. GO analysis of distinctively expressed sEV proteins in BC plasma samples using UCT

CATEGORY	TERM	COUNT	%	PVALUE	GENES	FDR
KEGG_PATHWAY	Complement and coagulation cascades	4	57.1428571	4.41E-06	VTN, CFI, F13A1, C4BPA	5.74E-05
REACTOME_PATHWAY	Regulation of Complement cascade	3	42.8571429	1.79E-04	VTN, CFI, C4BPA	0.00423363
REACTOME_PATHWAY	Complement cascade	3	42.8571429	2.73E-04	VTN, CFI, C4BPA	0.00423363
REACTOME_PATHWAY	Immune System	5	71.4285714	0.0049698	VTN, CFI, F13A1, C4BPA, PRSS3	0.05135459
REACTOME_PATHWAY	Innate Immune System	4	57.1428571	0.00798631	VTN, CFI, C4BPA, PRSS3	0.06189393

Table S7. GO analysis of distinctively expressed sEV proteins in BC plasma samples using UC.

CATEGORY	TERM	COUNT	%	PVALUE	GENES	FDR
REACTOME_PATHWAY	Platelet degranulation	8	24.2424242	1.08E-08	TF, VWF, IGF2, FN1, FLNA, TAGLN2, A2M, THBS1	2.02E-06
REACTOME_PATHWAY	Response to elevated platelet cytosolic Ca2+	8	24.2424242	1.41E-08	TF, VWF, IGF2, FN1, FLNA, TAGLN2, A2M, THBS1	2.02E-06
REACTOME_PATHWAY	Hemostasis	11	33.3333333	4.83E-07	SPN, TF, VWF, IGF2, FN1, FLNA, TAGLN2, A2M, THBS1, KLKB1, PF4V1	4.62E-05
REACTOME_PATHWAY	Platelet activation, signalling and aggregation	8	24.2424242	1.42E-06	TF, VWF, IGF2, FN1, FLNA, TAGLN2, A2M, THBS1	1.02E-04
REACTOME_PATHWAY	Regulation of Insulin-like Growth Factor (IGF) transport and uptake by Insulin-like Growth Factor Binding Proteins (IGFBPs)	6	18.1818182	7.93E-06	C3, TF, IGF2, FN1, APOL1, KTN1	4.55E-04
KEGG_PATHWAY	Complement and	5	15.1515152	5.88E-05	C3, VWF, C9, A2M, KLKB1	0.00370256

	coagulation cascades					
REACTOME_PATHWAY	Formation of Fibrin Clot (Clotting Cascade)	4	12.1212121	9.11E-05	VWF, A2M, KLKB1, PF4V1	0.00396236
REACTOME_PATHWAY	Post-translational protein phosphorylation	5	15.1515152	9.66E-05	C3, TF, FN1, APOL1, KTN1	0.00396236
KEGG_PATHWAY	Focal adhesion	6	18.1818182	1.24E-04	COMP, VWF, FN1, ZYX, FLNA, THBS1	0.00390762
REACTOME_PATHWAY	Extracellular matrix organization	6	18.1818182	5.06E-04	COMP, VWF, FN1, A2M, THBS1, KLKB1	0.01815743
REACTOME_PATHWAY	Integrin cell surface interactions	4	12.1212121	9.19E-04	COMP, VWF, FN1, THBS1	0.02929698
REACTOME_PATHWAY	Intrinsic Pathway of Fibrin Clot Formation	3	9.09090909	0.00123035	VWF, A2M, KLKB1	0.03531094
KEGG_PATHWAY	ECM-receptor interaction	4	12.1212121	0.0013788	COMP, VWF, FN1, THBS1	0.02895486
REACTOME_PATHWAY	Complement cascade	3	9.09090909	0.00765519	C3, FCN3, C9	0.19973096
KEGG_PATHWAY	signalling pathway	5	15.1515152	0.01138174	COMP, VWF, IGF2, FN1, THBS1	0.17926233
KEGG_PATHWAY	Proteoglycans in cancer	4	12.1212121	0.01466886	IGF2, FN1, FLNA, THBS1	0.18482767

REACTOME_PATHWAY	GP1b-IX-V activation signalling	2	6.06060606	0.02707904	VWF, FLNA	0.55149347
REACTOME_PATHWAY	Scavenging of heme from plasma	2	6.06060606	0.02930357	HP, APOL1	0.55149347
REACTOME_PATHWAY	GRB2:SOS provides linkage to MAPK signalling for Integrins	2	6.06060606	0.03373799	VWF, FN1	0.55149347
REACTOME_PATHWAY	p130Cas linkage to MAPK signalling for integrins	2	6.06060606	0.03373799	VWF, FN1	0.55149347
REACTOME_PATHWAY	Diseases of hemostasis	2	6.06060606	0.0359479	VWF, KLKB1	0.55149347
REACTOME_PATHWAY	Defects of contact activation system (CAS) and kallikrein/kinin system (KKS)	2	6.06060606	0.0359479	VWF, KLKB1	0.55149347
REACTOME_PATHWAY	Post-translational protein modification	8	24.2424242	0.03793785	C3, TF, PSMA1, DCTN2, FN1, APOL1, THBS1, KTN1	0.55149347
REACTOME_PATHWAY	Cell surface interactions at the vascular wall	3	9.09090909	0.0386652	SPN, FN1, PF4V1	0.55149347

REACTOME_PATHWAY	Degradation of the extracellular matrix	3	9.09090909	0.04021658	FN1, A2M, KLKB1	0.55149347
REACTOME_PATHWAY	Cell-extracellular matrix interactions	2	6.06060606	0.04035318	FLNA, LIMS1	0.55149347
KEGG_PATHWAY	hsa05165:Human papillomavirus infection	4	12.1212121	0.05051978	COMP, VWF, FN1, THBS1	0.51205955
REACTOME_PATHWAY	Initial triggering of complement	2	6.06060606	0.05128199	C3, FCN3	0.66899686
KEGG_PATHWAY	Phagosome	3	9.09090909	0.05689551	C3, COMP, THBS1	0.51205955
REACTOME_PATHWAY	Syndecan interactions	2	6.06060606	0.05993891	FN1, THBS1	0.71676945
REACTOME_PATHWAY	Integrin signalling	2	6.06060606	0.05993891	VWF, FN1	0.71676945
REACTOME_PATHWAY	Metabolism of proteins	9	27.2727273	0.06580889	C3, TF, PSMA1, DCTN2, IGF2, FN1, APOL1, THBS1, KTN1	0.75548611
REACTOME_PATHWAY	Immune System	9	27.2727273	0.07515349	C3, PIGR, FCN3, PSMA1, DCTN2, C9, HP, FN1, FLNA	0.79195136
REACTOME_PATHWAY	Signalling by high-kinase activity BRAF mutants	2	6.06060606	0.07914058	VWF, FN1	0.79195136

REACTOME_PATHWAY	Platelet Aggregation (Plug Formation)	2	6.06060606	0.08545704	VWF, FN1	0.79195136
REACTOME_PATHWAY	MAP2K and MAPK activation	2	6.06060606	0.08755327	VWF, FN1	0.79195136
REACTOME_PATHWAY	~Innate Immune System	6	18.1818182	0.0903635	C3, PIGR, FCN3, PSMA1, C9, HP	0.79195136
REACTOME_PATHWAY	Binding and Uptake of Ligands by Scavenger Receptors	2	6.06060606	0.0917319	HP, APOL1	0.79195136
REACTOME_PATHWAY	Signalling by RAF1 mutants	2	6.06060606	0.09381432	VWF, FN1	0.79195136

Table S8. Potential sEV protein biomarker isolated by UC method.

POTENTIAL SEV BIOMARKERS	EXPRESSION IN BC PLASMA	ABUNDANCE RATIO (LOG2): BC/NON-CANCER	P VALUE
**KRT10**	Upregulation	1.44	0.046483305
**STOM**	Upregulation	2.22	0.010063307
**F2**	Upregulation	2.16	0.012802556
**F5**	Upregulation	2.11	0.027789899
**TUBB1**	Upregulation	2.49	0.041497799
**HPR**	Upregulation	3.87	0.034695762

**ITIH1**	Upregulation	2.83	0.033002719
**GP1BB**	Upregulation	1.12	0.021107319
**IGHA1**	Upregulation	4.65	0.000467739
**IGHG4**	Upregulation	2.19	0.001960026
**LPA**	Upregulation	3	0.029432023
**APOA4**	Downregulation	-3.31	0.004124821
**APOE**	Downregulation	-1.97	0.005521731
**CSN1S1**	Downregulation	-0.9	0.005749587

Table S9. Potential sEV protein biomarker isolated by UCT method.

POTENTIAL SEV BIOMARKERS	EXPRESSION IN BC PLASMA	ABUNDANCE RATIO (LOG2): BC/NON-CANCER	P VALUE
**AHSG**	Upregulation	2.34	0.030585
**ALB**	Upregulation	3.25	0.022753
**COL1A1**	Upregulation	2.97	0.030104
**CSN1S1**	Upregulation	2.55	0.031649
		3.06	0.02753
		2.78	0.03789
		3.62	0.033101
**EEF1A2**	Upregulation	5.32	0.013567
**KRT9**	Upregulation	1.75	0.031303

**RPL3**	Upregulation	2.54	0.019181
**APOA4**	Downregulation	-2.37	0.01328465
**APOC1**	Downregulation	-2.7	0.04784891
**C1S**	Downregulation	-4.54	0.00111333
**C3**	Downregulation	-2.45	0.01899849
**C5**	Downregulation	-4.47	0.04643546
**C7**	Downregulation	-2.88	0.00067045
**C8G**	Downregulation	-1.55	0.00031382
**CFHR2**	Downregulation	-3.48	0.03053928
**CLU**	Downregulation	-1.52	0.01986562
**FGA**	Downregulation	-1.5	0.03166746
**FGG**	Downregulation	-2.63	0.02426461
**ITIH1**	Downregulation	-1.98	0.00189802
**KLKB1**	Downregulation	-2.05	0.03297687
**RBP4**	Downregulation	-0.94	0.04068649
**SERPINC1**	Downregulation	-1.82	0.01121957
**TTR**	Downregulation	-1.37	0.03719059
